# Real‐time intrafraction prostate motion during linac based stereotactic radiotherapy with rectal displacement

**DOI:** 10.1002/acm2.12195

**Published:** 2017-09-27

**Authors:** Kimberley Legge, Doan Nguyen, Jin Aun Ng, Lee Wilton, Matthew Richardson, Jeremy Booth, Paul Keall, Darryl J O'Connor, Peter Greer, Jarad Martin

**Affiliations:** ^1^ School of Mathematical and Physical Sciences University of Newcastle Callaghan NSW Australia; ^2^ Radiation Physics Laboratory University of Sydney Sydney NSW Australia; ^3^ Radiation Oncology Department Calvary Mater Newcastle Newcastle NSW Australia; ^4^ Northern Sydney Cancer Centre Royal North Shore Hospital St Leonards NSW Australia; ^5^ School of Mathematical and Physical Sciences University of Newcastle Callaghan NSW Australia; ^6^ Radiation Oncology Department Calvary Mater Newcastle Newcastle NSW Australia; ^7^ Hunter Region Mail Centre Calvary Mater Newcastle Newcastle NSW Australia

**Keywords:** intrafraction motion, stereotactic radiation therapy

## Abstract

**Background:**

Kilovoltage Intrafraction Monitoring (KIM) is a method which determines the three‐dimensional position of the prostate from two‐dimensional kilovoltage (kV) projections taken during linac based radiotherapy treatment with real‐time feedback. Rectal displacement devices (RDDs) allow for improved rectal dosimetry during prostate cancer treatment. This study used KIM to perform a preliminary investigation of prostate intrafraction motion observed in patients with an RDD in place.

**Methods:**

Ten patients with intermediate to high‐risk prostate cancer were treated with a Rectafix RDD in place during two boost fractions of 9.5–10 Gy delivered using volumetric modulated arc therapy (VMAT) on Clinac iX and Truebeam linacs. Two‐dimensional kV projections were acquired during treatment. KIM software was used following treatment to determine the displacement of the prostate over time. The displacement results were analyzed to determine the percentage of treatment time the prostate spent within 1 mm, between 1 and 2 mm, between 2 and 3 mm and greater than 3 mm from its initial position.

**Results:**

KIM successfully measured displacement for 19 prostate stereotactic boost fractions. The prostate was within 1 mm of its initial position for 84.8%, 1–2 mm for 14%, 2–3 mm 1.2% and ≥3 mm only 0.4% of the treatment time.

**Conclusions:**

In this preliminary study using KIM, KIM was successfully used to measure prostate intrafraction motion, which was found to be small in the presence of a rectal displacement device.

**Trial registration:**

The Hunter New England Human Research Ethics Committee reference number is 14/08/20/3.01.

## 1 BACKGROUND

**Figure 1:**
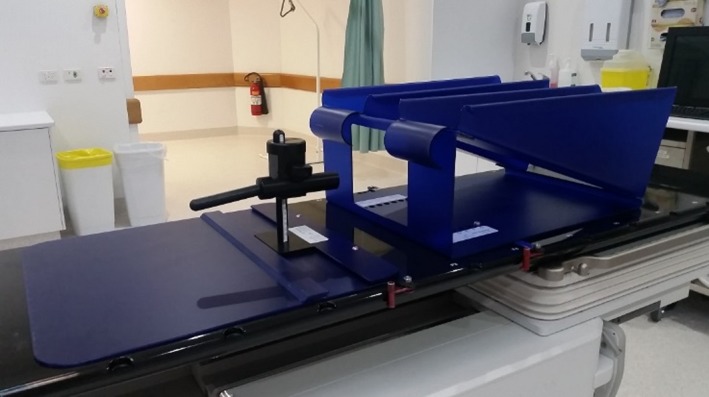
The Rectafix system. The system consists of a rectal retracting rod attached to a vertical column and locked onto a baseplate. A leg rest is also provided.

Prostate cancer is estimated to have a low *α*/*β* ratio, indicating that hypofractionated treatment schedules may increase the effectiveness of treatment.^1^ Delivery of higher doses through hypofractionation increases the risk of damage to healthy tissues surrounding the prostate, particularly the rectal wall.^2^ The risk and severity of rectal toxicities have been correlated with the volume of rectal wall exposed to high doses of radiation.^3^ The application of dose volume constraints in planning, along with daily image guidance to enable reduced margins, are the most effective ways to reduce rectal dose, but rectal displacement devices such as injected hydrogel and the Rectafix (Scanflex Medical AB, Tumstocksvägen, Sweden) rectal retractor are also useful in allowing for safe dose escalation.^4^ The Rectafix system (Fig. 1) uses a rod inserted into the patient's rectum and then gently depressed posteriorly and fixed in place, guided by patient tolerance, thereby manually moving the rectum away from the prostate. The Rectafix provides an average increase in separation of 0.5 cm between the anterior rectal wall and posterior prostate border, and may assist in immobilizing the rectal wall by preventing changes in filling by gas or feces.^5^

Intrafraction prostate motion has the potential to reduce the dose coverage of the prostate and to increase the dose received by organs at risk. A variety of methods exist for monitoring the position of the prostate during treatment, including megavoltage (MV) imaging,^6^ ultrasound,^7^ combined MV and kilovoltage (kV) imaging,^8^ Calypso electromagnetic guidance,^9^ the BrainLAB ExacTrac x‐ray system,^10^ the Cyberknife platform,^11^, ^12^, ^13^ and Navotek radioactive fiducials.^14^ Several of these methods require additional equipment not available on a standard linear accelerator, are costly, and require further expertise to implement.

Kilovoltage Intrafraction Monitoring (KIM) takes advantage of the gantry‐mounted kV imager available on many modern linear accelerators to determine the position of the prostate in three dimensions from 2D kV projections using a probability density function.^15^, ^16^ The geometrical accuracy of KIM has been established, and the software has successfully been used to measure prostate displacement during treatment in noninterventional^17^ and interventional studies.^18^

In this study, KIM was used to perform a preliminary investigation of the magnitude of intrafraction motion in a series of clinical trial patients receiving Stereotactic Body Radiation Therapy (SBRT) to the prostate with a Rectafix in place.

## 2 METHODS

### 2.A Quality assurance of KIM software

The KIM software was used offline to analyze kV images acquired during delivery to determine prostate motions. Quality assurance (QA) of the KIM software was performed on the Varian Clinac iX machine.

The tests used were those suggested for KIM QA by Ng et al,^19^ which were based on the recommendations for Calypso QA by Santanam et al^20^ The tests suggested were a static localisation test, a dynamic test, a latency test, and a treatment interruption test. The latency test and treatment interruption test were not performed as KIM software was not intended for real‐time use or gating during this study. The pass criteria applied for both the static and dynamic tests was 1.0 mm mean difference and 1.0 mm standard deviation between the KIM software trajectory and the trajectory output by the motion phantom.

Static localisation tests were performed to ensure that KIM was able to trace static offsets correctly and that all directions of the software and phantom coordinate systems were in agreement. Static localisation tests were performed using the CIRS 801‐P Virtually Human Male Pelvis phantom (CIRS Inc., Norfolk, VA, USA) with an insert containing three cylindrical gold fiducial markers with dimensions 0.9 × 3 mm. The phantom was offset ±5 mm in each of the anterior‐posterior (AP), left‐right (LR), and superior‐inferior (SI) directions. A pre‐arc of images taken during a rotation of 120° prior to treatment commencement and one partial treatment arc were delivered to the phantom at each position while kV images were acquired at 10 Hz. The position determined by KIM was compared to the known static shift and the mean difference and standard deviation were determined.

For dynamic localisation tests, a pelvic Rando phantom (The Phantom Laboratory, Salem, NY) with three implanted cylindrical fiducial markers with dimensions 1.0 × 3.0 mm was placed on a custom‐made platform attached to a HexaMotion (Scandidos, Uppsala, Sweden) motion phantom. The HexaMotion was programmed with six realistic prostate motion trajectories – stable, continuous drift, persistent excursion, transient excursion, high‐frequency excursion, and erratic behavior. One treatment arc with kV images acquired at 10 Hz was delivered for each of the motion trajectories programmed to the HexaMotion. The mean difference and standard deviation between the programmed and KIM measured trajectory were determined for comparison.

### 2.B Patient and treatment details

The PROstate Multicentre External beam radioTHErapy Using Stereotactic boost (PROMETHEUS) clinical trial is a hypofractionated boost study delivering a stereotactic boost dose consisting of two 9.5–10 Gy fractions to patients prior to a 46 Gy course of conventionally fractionated external beam radiation therapy (EBRT).

Ten patients with intermediate to high‐risk prostate cancer (median age 72) provided informed consent to participate in the trial. Patients each had three cylindrical gold fiducial markers (3 mm long, 0.9 mm diameter) inserted into their prostate. Seven patients (patients 1 to 7) received a boost dose of 9.5 Gy per fraction and three patients (patients 8 to 10) received a boost dose of 10 Gy per fraction. A PTV margin of 5 mm was applied in all directions, except posteriorly where the margin was 3 mm. Patients had a Rectafix device inserted during simulation CT and boost fractions to increase the posterior displacement of the rectal wall. Planning CT scans were acquired with a 1 mm slice thickness. All patients were instructed to be nil by mouth from the night prior to simulation and treatment with only clear fluids permitted. Patients were booked to receive early appointments to limit gastrointestinal activation. Patients took Benefibre in the fortnight prior to treatment, and were given a self‐administered Microlax enema and emptied their bowels and bladder 1 hour before treatment. Patients consumed one cup of water 30 min before the commencement of treatment.

Three patients (patients 1 to 3) were treated on a Varian Clinac iX linear accelerator (6X, maximum dose rate of 600 MU/min), and seven patients (patients 4 to 10) were treated on a Varian Truebeam linear accelerator (one, patient 5, with 10X, maximum dose rate of 600 MU/min, and six with 10X flattening filter free (FFF), maximum dose rate of 2400 MU/min). All patients were treated using a two partial arc VMAT technique, during which kV images were acquired. Data was acquired for a total of 20 patient boost fractions. Treatments using a maximum dose rate of 600 MU/min had an average beam on time of 364 ± 67 s, and treatments using a maximum dose rate of 2400 MU/min had an average beam on time of 93 ± 6 s.

### 2.C IGRT and KIM acquisition

Patients treated on the Clinac were first aligned using cone beam computed tomography (CBCT). Two‐dimensional kV images were then acquired using the kV imager mounted on the gantry perpendicular to the treatment beam. Images were taken at 125 kV, 80 mA and 13 ms at 5 Hz with a 6 × 6 cm field size and 180 cm imager source to detector distance (SDD). The 180 cm SDD decreases the effect of MV scatter on the kV images. Images were taken over a gantry rotation of 120° immediately prior to treatment, and then as the gantry rotated during delivery of VMAT treatment. This 120° pre‐arc was necessary to allow an earlier version of the KIM software to build its probability density model to track the fiducial marker positions. KIM software has since been updated so that pre‐treatment CBCT imaging can instead be used to build the model. Imaging during treatment was enabled using the service mode of the on‐board imaging software, which is not currently possible in the clinical mode. Images were saved using a research framegrabber computer and in‐house software. No real‐time IGRT was used for these treatments.

Patients treated on the Truebeam were first aligned using kV/kV matching to assess for gross RDD error or bowel gas. A full fan spotlight CBCT was then acquired for further alignment purposes and to enable the KIM software to build its probability density function. Images were acquired during delivery of VMAT treatment at 0.33 Hz with a 125 kV, 80 mA and 13 ms beam. A field size of 5 × 5 cm and an imager SDD of 180 cm was used for these patients. At the time of treatment, KIM was unable to be used in real time on the Truebeam platform. Instead, the clinical intrafraction motion review software on the Truebeam was used to monitor the real‐time prostate location during delivery. This software generates a DRR at the same angle as the acquired kV image and compares beam's‐eye‐view position of the marker segmented on the image and the DRR marker position. A tolerance for agreement of the position of each marker can be set. A tolerance circle of 2 mm radius was used with manual treatment interruption by the radiation therapist. Kilovoltage images were acquired every 3 s during treatment delivery, as this is the fastest imaging frequency available which allows for use of the Truebeam clinical intrafraction motion review software. The field size was reduced to 5 × 5 cm for patients treated on the Truebeam, as it provided better marker visibility within the Truebeam clinical intrafraction motion review software. All CBCT and kilovoltage images were saved and fed into the KIM software offline following treatment.

### 2.D Analysis

Following treatment, the pre‐arc/CBCT images and the kV projections taken during treatment were processed offline using the KIM software. The KIM software automatically segments the location of each fiducial marker on each 2D kV projection, then reconstructs the 3D position of the markers by taking a maximum likelihood estimation of a 3D probability density function.^21^ The displacement of the prostate in each of the AP, LR, and SI directions was quantified as a function of time throughout each fraction.

## 3 RESULTS

### 3.A Quality assurance of KIM software

The results of both the static and dynamic localisation tests appear in Table 1, which shows the mean difference ($\bar{x}$) and standard deviation (σ) between the expected position of the phantom and the KIM measured position. All static localisation tests passed the criteria of <1 mm mean difference and <1 mm standard deviation. All dynamic tests passed the criteria of <1 mm mean difference and <1 mm standard deviation, apart from the high frequency and erratic trajectories, which both failed due to a standard deviation greater than 1 mm in the AP direction. These two motion trajectories represent the most extreme prostate motion, with high frequency, high amplitude motion, and are, therefore, the most difficult traces for KIM to track accurately.

**Table 1:** Static and dynamic localization results for QA of KIM software

Phantom position or trajectory	AP		LR		SI	
	$\bar{x}$ (mm)	σ (mm)	$\bar{x}$ (mm)	σ (mm)	$\bar{x}$ (mm)	σ (mm)
−5 mm LR	−0.66	0.26	−0.28	0.31	0.13	0.15
+5 mm LR	−0.68	0.25	−0.22	0.37	0.10	0.17
−5 mm SI	0.01	0.24	0.17	0.32	−0.48	0.16
+5 mm SI	0.60	0.28	−0.20	0.45	−0.31	0.16
−5 mm AP	−0.08	0.24	0.19	0.39	−0.48	0.16
+5 mm AP	0.38	0.23	0.15	0.36	−0.46	0.17
Stable	−0.27	0.30	0.41	0.29	−0.03	0.19
Continuous Drift	0.24	0.38	0.15	0.55	−0.01	0.18
Persistent Excursion	−0.56	0.57	0.09	0.22	−0.00	0.23
Transient Excursion	−0.27	0.54	−0.04	0.23	−0.00	0.18
High Frequency	−0.58	1.72	−0.05	0.53	−0.06	0.95
Erratic	0.24	1.18	0.44	0.64	0.02	0.27

### 3.B Patient motion results

The KIM software gives displacement of the prostate in the AP, LR, and SI directions as a function of time throughout each fraction. These results were analyzed to determine the percentage of time the prostate spent within 1 mm, between 1 and 2 mm, between 2 and 3 mm, and ≥3 mm from its initial position in each of the three directions. These results appear in Table 2. Table 3 displays the average and standard deviation for prostate displacement across all patients and fractions measured.

**Table 2:** Prostate displacement by direction and distance as a percentage of treatment time

Patient	Direction	% of Displacement			
		<1 mm	1–2 mm	2–3 mm	≥3 mm
1	AP	36.8	33.5	18.1	11.7
	LR	91.0	9.0	0.0	0.0
	SI	95.7	4.1	0.1	0.1
2	AP	69.7	30.2	0.1	0.0
	LR	73.4	26.6	0.0	0.0
	SI	99.7	0.2	0.1	0.0
3	AP	59.0	35.7	5.2	0.1
	LR	66.3	33.7	0.0	0.0
	SI	99.8	0.2	0.0	0.0
4	AP	94.2	5.8	0.0	0.0
	LR	94.9	5.1	0.0	0.0
	SI	100.0	0.0	0.0	0.0
5	AP	95.4	4.2	0.4	0.0
	LR	94.1	5.9	0.0	0.0
	SI	100.0	0.0	0.0	0.0
6	AP	79.0	21.0	0.0	0.0
	LR	85.5	14.5	0.0	0.0
	SI	90.3	9.7	0.0	0.0
7	AP	65.2	34.8	0.0	0.0
	LR	98.5	1.5	0.0	0.0
	SI	97.0	3.0	0.0	0.0
8	AP	84.2	15.8	0.0	0.0
	LR	54.2	25.0	16.7	4.1
	SI	91.2	8.8	0.0	0.0
9	AP	98.3	1.7	0.0	0.0
	LR	67.7	29.0	3.3	0.0
	SI	76.7	23.3	0.0	0.0
10	AP	76.8	23.2	0.0	0.0
	LR	88.7	11.3	0.0	0.0
	SI	100.0	0.0	0.0	0.0
All Patients	AP	79.1	18.2	1.1	0.8
	LR	82.7	15.8	0.9	0.3
	SI	97.2	2.8	0.0	0.0
	Overall	84.8	13.6	0.8	0.4

**Table 3:** Average prostate displacement in each direction for all patients across all fractions measured

Direction	Average (mm)	STD (mm)	Average (Directionless) (mm)
AP	0.11	0.64	0.61
LR	0.02	0.23	0.18
SI	−0.21	0.12	0.21

The majority of motion measured by the KIM system occurred in the AP and LR directions. The prostate was greater than 1 mm from its initial position in the SI direction only 2.8% of the treatment time (see Table 2); however, the average displacement was greatest in the SI direction, while still being sub‐millimeter (see Table 3).

**Figure 2:**
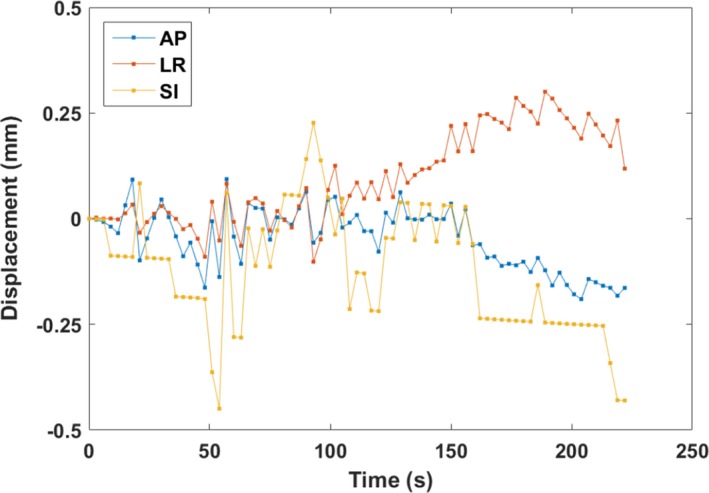
The most stable prostate trajectory recorded during the study. Motion in the AP, LR, and SI directions is plotted as a function of time.

Figure 2 shows an extremely stable prostate trajectory observed during this study, plotted as a function of time. Prostate motion during this arc did not exceed 0.5 mm in any direction.

**Figure 3:**
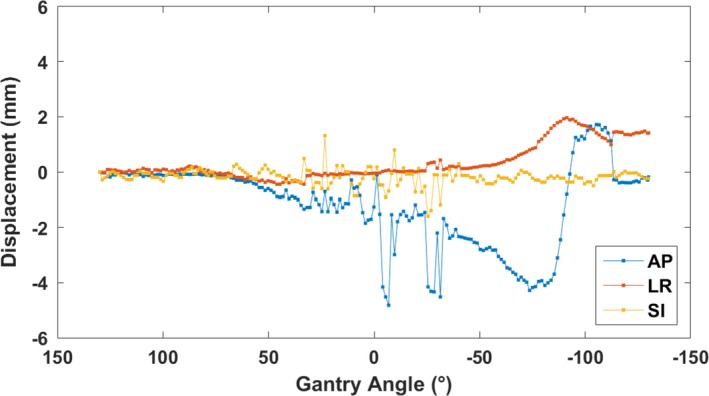
The least stable prostate displacement recorded during the study. Motion in the AP, LR, and SI directions is plotted as a function of gantry angle for a single prostate SBRT boost treatment to highlight segmentation issues occurring when the kV projections are being taken through the widest part of the patient when the gantry is at zero degrees.

Figure 3 shows a prostate motion trajectory for a single patient treatment plotted in the AP, LR, and SI directions as a function of gantry angle. The displacements towards the end of this trajectory were due to a gas bubble descending into the patient's rectum which was observed on post‐treatment CBCT. This trajectory was the most unstable trajectory recorded during this study.

KIM was able to segment markers in the majority of images; however, for patients treated on Clinac, image quality was low when the images were being acquired across the widest part of the hip (around gantry 0°), resulting in errors in seed segmentation. The large, rapidly changing displacement values around gantry 0° in Fig. 3 are a result of erroneous segmentation in extremely low‐quality images.

## 4 DISCUSSION AND CONCLUSIONS

The KIM software was successfully used to segment fiducial markers and determine prostate motion during prostate SBRT boost treatments in 95% of fractions for ten patients with a Rectafix in place. As such, it would appear to be a feasible approach to deploy clinically on either a Clinac or TrueBeam linear accelerator. KIM performs better when the incoming images have higher image quality — this was observed for patients with a smaller distance across the hips treated on the Clinac, and for all patients treated on the Truebeam.

Very little motion was observed in this patient cohort. However, these results should be considered preliminary and further work with larger patient cohorts and homogeneous image acquisition and treatment is required. This should enable detailed examination of changes in observed motion due to reduced treatment times, patient preparation techniques, and rectal immobilization. To fully separate the effect of the Rectafix on prostate motion would require a randomized study design with a comparison to patients without Rectafix. However, this study has demonstrated that the implementation of KIM imaging for SBRT prostate intrafraction motion measurement is feasible in the clinic and can be utilized to assess new clinical practices including rectal immobilization techniques. The prostate was two or more millimeters from its initial position only 1.2% of the time. Patients 1 to 3 treated on the Clinac showed a higher percentage of prostate displacement over 1 mm than patients 4 to 7 treated on the Truebeam. The majority of motion observed occurred in the AP and LR directions, with very little motion observed in the SI direction. This finding corresponds with the results of De Leon et al,^22^ who found in a study of cine‐MRI images for patients with and without a Rectafix that the presence of the Rectafix significantly reduced prostate displacement in the anterior‐posterior and superior‐inferior directions.^22^ This is in contrast to the studies on intrafraction prostate motion in patients without a Rectafix discussed below, which show minimal motion in the LR direction and greater levels of motion in the SI and AP directions.

Less motion was observed in the patient cohort considered here, treated with a Rectafix in place, when compared with the data reported by Ng et al^17^ who found, in a study of 10 patients without an RDD, that the prostate was displaced less than 1 mm from its initial position only 62.4% of the time, and was more than 3 mm from its initial position 4.7% of the time. Keall et al^23^ reported on the use of KIM in an interventional study for 197 prostate fractions delivered to 6 patients using VMAT, where, if no repositioning had occurred, the prostate would have been greater than 3 mm from its isocentre position for 20% of the beam on treatment time and greater than 5 mm from the isocentre position for 4% of the treatment time. In the patient cohort measured here, the prostate was greater than 3 mm from its initial position for less than 1% of the treatment time. The Calypso transponder system was used by Su et al^24^ to monitor prostate motion for an average of 28 fractions per patient for 17 patients treated without a RDD in place. The 3D displacement of the prostate was found to be greater than 3 mm for approximately 20% of the total monitoring time, and greater than 5 mm for about 9% of the treatment time, again demonstrating a much larger level of motion than that observed in patients treated with a Rectafix in place considered in this study. The results reported in this paper are in contrast to those of Vanhanen and Kapanen^25^ who found increased prostate motion during treatment fractions with a RDD in position compared to fractions without a RDD. Patients in that study were only instructed to have an empty rectum and full bladder prior to treatment, whereas in this study patients had a far stricter preparation regime prior to treatments. It is proposed that the difference in results is due to the difference in bowel and bladder preparation regimes applied during each study.

There are several limitations in this study. Due to the Truebeam becoming available for clinical use during the study, the clinical decision was made to transfer patients to this machine to enable use of higher energy and higher dose rate for treatments. For this reason, only three patients were treated on the Clinac iX machine, with the rest being treated on Truebeam. The image guidance strategies were different on these two machines with real time on the Truebeam, using the clinical intrafraction motion review software, and inter‐arc on the Clinac. Any influence of these different strategies has been minimized by removing the positional changes from the data and presenting the prostate displacement in each direction.

The low imaging frequency of 0.33 Hz used on Truebeam increases the uncertainty in the results, as sub‐millimeter accuracy of the software has only been tested for imaging frequencies down to 1 Hz.^26^ This frequency of imaging was selected so that real‐time positional feedback through the Varian Truebeam intrafraction motion review was available during treatment. The patients treated on Truebeam were treated before the KIM system was able to run on the Truebeam platform in real time. Further development of the KIM software and the in‐house image acquisition software has since enabled KIM to function in real time on the Truebeam platform with fluoroscopic x‐ray imaging.

In Fig. 3, the displacements exceeding 2 mm where the gantry angle is close to 0° are due to poor image quality, resulting in KIM failing to segment seeds correctly. Studies using KIM generally impose a maximum hip width of 40 cm for patient inclusion to increase image quality. This patient's hip width was the widest in the study at 40.2 cm, but the patient was included anyway as the study was noninterventional.

The minimal motion observed in patients treated with a Rectafix device combined with a strict preparatory regime may allow the future reduction of PTV margins for patients with an RDD in place, as the minimized target motion resulting from the use of the Rectafix device will help to ensure adequate dose coverage. This has important implications for increasing the safety and efficacy of hypofractionated prostate radiation therapy schedules, as the reduction of margins combined with the protection of the rectum provided by the RDD will allow greater dose escalation without increasing dose to the organs at risk. The preliminary data shown here suggest that intrafraction motion monitoring may not be necessary for patients with a Rectafix device in place, however, further work is needed with larger patient cohorts to confirm this finding. Bowel and bladder preparation combined with pre‐treatment and mid‐treatment imaging to assist positioning and assess bladder and rectal fullness is likely to be sufficient, as such small displacements were observed in this cohort so that the additional imaging dose provided by kV imaging can be avoided. Future work in this area is required to determine if the motion reduction is due to the Rectafix alone, or if the pre‐treatment bowel and bladder preparation regime followed by patients or fast treatment times has the most impact on intrafraction prostate motion.

Current work being performed in relation to the PROMETHEUS study includes an investigation of patient tolerance of the Rectafix device via patient questionnaires. A dosimetric comparison of the SpaceOAR rectal displacement system and the Rectafix rectal retractor has also commenced.

Intrafraction motion results obtained from both KIM software and pre‐ and post‐treatment couch positions for patients receiving prostate SBRT boost treatments with a Rectafix device in place show considerably less prostate displacement during treatment when compared with patients treated without a Rectafix device in place. The prostate was two or more millimeters from its initial position only 1.2% of the time. This may have implications regarding appropriate CTV‐PTV margins, tumor dose delivery, and normal tissue sparing.

## CONFLICT OF INTEREST

The authors declare that they have no conflicts of interest.

## References

[1] Williams SG , Taylor JM , Liu N . Use of individual fraction size data from 3756 patients to directly determine the alpha/beta ratio of prostate cancer. Int J Radiat Oncol Biol Phys. 2007;68:24–33. 17448868 10.1016/j.ijrobp.2006.12.036

[2] Xie Y , Djajaputra D , King CR , Hossain S , Ma L , Xing L . Intrafractional motion of the prostate during hypofractionated radiotherapy. Int J Radiat Oncol Biol Phys. 2008;72:236–246. 18722274 10.1016/j.ijrobp.2008.04.051 PMC2725181

[3] Hanks GE , Schultheiss TE , Hanlon AL , et al. Optimization of conformal radiation treatment of prostate cancer: report of a dose escalation study. Int J Radiat Oncol Biol Phys. 1997;37:543–550. 9112451 10.1016/s0360-3016(96)00602-5

[4] Valdagni R , Rancati T . Reducing rectal injury during external beam radiotherapy for prostate cancer. Nat Rev Urol. 2013;10:345–357. 23670182 10.1038/nrurol.2013.96

[5] Isacsson U , Nilsson K , Asplund S , Morhed E , Montelius A , Turesson I . A method to separate the rectum from the prostate during proton beam radiotherapy of prostate cancer patients. Acta Oncol. 2010;49:500–505. 20397777 10.3109/02841861003745535

[6] Berbeco RI , Hacker F , Ionascu D , Mamon HJ . Clinical feasibility of using an EPID in cine mode for image‐guided verification of stereotactic body radiotherapy. Int J Radiat Oncol Biol Phys. 2007;69:258–266. 17707280 10.1016/j.ijrobp.2007.04.051

[7] Schlosser J , Salisbury K , Hristov D . Telerobotic system concept for real‐time soft‐tissue imaging during radiotherapy beam delivery. Med Phys. 2010;37:6357–6367. 21302793 10.1118/1.3515457

[8] Cho B , Poulsen PR , Sloutsky A , Sawant A , Keall PJ . First demonstration of combined kV/MV image‐guided real‐time dynamic multileaf‐collimator target tracking. Int J Radiat Oncol Biol Phys. 2009;74:859. 19480969 10.1016/j.ijrobp.2009.02.012 PMC2720135

[9] Kupelian P , Willoughby T , Mahadevan A , et al. Multi‐institutional clinical experience with the Calypso System in localization and continuous, real‐time monitoring of the prostate gland during external radiotherapy. Int J Radiat Oncol Biol Phys. 2007;67:1088–1098. 17187940 10.1016/j.ijrobp.2006.10.026

[10] Jin J‐Y , Yin F‐F , Tenn SE , Medin PM , Solberg TD . Use of the BrainLAB ExacTrac x‐ray 6D system in image‐guided radiotherapy. Med Dosim. 2008;33:124–134. 18456164 10.1016/j.meddos.2008.02.005

[11] Oermann EK , Slack RS , Hanscom HN , et al. A pilot study of intensity modulated radiation therapy with hypofractionated stereotactic body radiation therapy (SBRT) boost in the treatment of intermediate‐ to high‐risk prostate cancer. Technol Cancer Res Treat. 2010;9:453–462. 20815416 10.1177/153303461000900503

[12] Katz AJ , Santoro M , Ashley R , Diblasio F , Witten M . Stereotactic body radiotherapy as boost for organ‐confined prostate cancer. Technol Cancer Res Treat. 2010;9:575–582. 21070079 10.1177/153303461000900605

[13] Jabbari S , Weinberg VK , Kaprealian T , et al. Stereotactic body radiotherapy as monotherapy or post–external beam radiotherapy boost for prostate cancer: technique, early toxicity, and PSA response. Int J Radiat Oncol Biol Phys. 2012;82:228–234. 21183287 10.1016/j.ijrobp.2010.10.026

[14] Shchory T , Schifter D , Lichtman R , Neustadter D , Corn BW . Tracking accuracy of a real‐time fiducial tracking system for patient positioning and monitoring in radiation therapy. Int J Radiat Oncol Biol Phys. 2010;78:1227–1234. 20615628 10.1016/j.ijrobp.2010.01.067

[15] Poulsen PR , Cho B , Keall PJ . A method to estimate mean position, motion magnitude, motion correlation, and trajectory of a tumor from cone‐beam CT projections for image‐guided radiotherapy. Int J Radiat Oncol Biol Phys. 2008;72:1587. 19028282 10.1016/j.ijrobp.2008.07.037

[16] Poulsen PR , Cho B , Keall PJ . Real‐time prostate trajectory estimation with a single imager in arc radiotherapy: a simulation study. Phys Med Biol. 2009;54:4019. 19502704 10.1088/0031-9155/54/13/005

[17] Ng JA , Booth JT , Poulsen PR , et al. Kilovoltage intrafraction monitoring for prostate intensity modulated arc therapy: first clinical results. Int J Radiat Oncol Biol Phys. 2012;84:e655–e661. 22975613 10.1016/j.ijrobp.2012.07.2367 PMC5357433

[18] Keall PJ , Aun J , Ng R , et al. The first clinical treatment with kilovoltage intrafraction monitoring (KIM): a real‐time image guidance method. Med Phys. 2015;42:354–358. 25563275 10.1118/1.4904023

[19] Ng JA , Booth JT , O'Brien RT , et al. Quality assurance for the clinical implementation of kilovoltage intrafraction monitoring for prostate cancer VMAT. Med Phys. 2014;41:111712. 25370626 10.1118/1.4898119

[20] Santanam L , Noel C , Willoughby TR , et al. Quality assurance for clinical implementation of an electromagnetic tracking system. Med Phys. 2009;36:3477–3486. 19746781 10.1118/1.3158812

[21] Poulsen PR , Cho B , Langen K , Kupelian P , Keall PJ . Three‐dimensional prostate position estimation with a single x‐ray imager utilizing the spatial probability density. Phys Med Biol. 2008;53:4331. 18660559 10.1088/0031-9155/53/16/008

[22] De Leon J , Rivest‐Henault D , Keats S , et al. PV‐0328: Rectal immobilisation device in stereotactic prostate treatment: intrafraction motion and dosimetry. Radiotherapy and Oncology. 2016;119:S151–S152.

[23] Keall PJ , Ng JA , Juneja P , et al. Real‐time 3D image guidance using a standard LINAC: measured motion, accuracy, and precision of the first prospective clinical trial of kilovoltage intrafraction monitoring–guided gating for prostate cancer radiation therapy. Int J Radiat Oncol Biol Phys. 2016;94:1015–1021. 27026307 10.1016/j.ijrobp.2015.10.009

[24] Su Z , Zhang L , Murphy M , Williamson J . Analysis of prostate patient setup and tracking data: potential intervention strategies. Int J Radiat Oncol Biol Phys. 2011;81:880–887. 20934274 10.1016/j.ijrobp.2010.07.1978 PMC3020989

[25] Vanhanen A , Kapanen M . The effect of rectal retractor on intrafraction motion of the prostate. Biomed Phys Eng Express. 2016;2:035021.

[26] Poulsen PR , Cho B , Sawant A , Keall PJ . Implementation of a new method for dynamic multileaf collimator tracking of prostate motion in arc radiotherapy using a single kV imager. Int J Radiat Oncol Biol Phys. 2010;76:914. 19910138 10.1016/j.ijrobp.2009.06.073

